# Nanoscale changes in chromatin organization represent the initial steps of tumorigenesis: a transmission electron microscopy study

**DOI:** 10.1186/1471-2407-14-189

**Published:** 2014-03-14

**Authors:** Lusik Cherkezyan, Yolanda Stypula-Cyrus, Hariharan Subramanian, Craig White, Mart Dela Cruz, Ramesh K Wali, Michael J Goldberg, Laura K Bianchi, Hemant K Roy, Vadim Backman

**Affiliations:** 1 Department of Biomedical Engineering, Northwestern University, Evanston, Illinois 60208, USA; 2 Department of Medicine, Boston Medical Center, Boston, Massachusetts 02118, USA; 3 Department of Internal Medicine, NorthShore University HealthSystem, Evanston, Illinois 60201, USA

**Keywords:** Chromatin, Colon cancer, Field cancerization, Field effect, Transmission electron microcopy

## Abstract

**Background:**

Nuclear alterations are a well-known manifestation of cancer. However, little is known about the early, microscopically-undetectable stages of malignant transformation. Based on the phenomenon of field cancerization, the tissue in the field of a tumor can be used to identify and study the initiating events of carcinogenesis. Morphological changes in nuclear organization have been implicated in the field of colorectal cancer (CRC), and we hypothesize that characterization of chromatin alterations in the early stages of CRC will provide insight into cancer progression, as well as serve as a biomarker for early detection, risk stratification and prevention.

**Methods:**

For this study we used transmission electron microscopy (TEM) images of nuclei harboring pre-neoplastic CRC alterations in two models: a carcinogen-treated animal model of early CRC, and microscopically normal-appearing tissue in the field of human CRC. We quantify the chromatin arrangement using approaches with two levels of complexity: 1) binary, where chromatin is separated into areas of dense heterochromatin and loose euchromatin, and 2) grey-scale, where the statistics of continuous mass-density distribution within the nucleus is quantified by its spatial correlation function.

**Results:**

We established an increase in heterochromatin content and clump size, as well as a loss of its characteristic peripheral positioning in microscopically normal pre-neoplastic cell nuclei. Additionally, the analysis of chromatin density showed that its spatial distribution is altered from a fractal to a stretched exponential.

**Conclusions:**

We characterize quantitatively and qualitatively the nanoscale structural alterations preceding cancer development, which may allow for the establishment of promising new biomarkers for cancer risk stratification and diagnosis. The findings of this study confirm that ultrastructural changes of chromatin in field carcinogenesis represent early neoplastic events leading to the development of well-documented, microscopically detectable hallmarks of cancer.

## Background

Chromatin arrangement has been extensively studied as it defines the physical and biochemical forces that govern genome function. Alterations in higher-order chromatin structure are associated with changes in gene expression, observed in many complex human diseases [1,2]. In particular, cells undergoing neoplastic transformation are characterized by the coarse, asymmetric aggregation of densely packed chromatin [3]. The tumorigenic changes in chromatin texture have been shown to be independent of cell-cycle progression [4]. Through chromatin remodeling mechanisms, genetic/epigenetic alterations of tumor suppressor genes or proto-oncogenes initiate and advance neoplastic progression. As a consequence, there is a large body of literature devoted to the structural differences between normal and cancerous cell nuclei [3,5]. However, the process of malignant alterations a nucleus undergoes at the earliest stages of carcinogenesis remains unclear.

The phenomenon of field cancerization (also known as field carcinogenesis, field effect, or field defect) is the concept that the altered genetic/epigenetic environment that gives rise to a focal tumor is present throughout the organ. That is, the aberrant genetic and environmental modifications create a fertile background on which individual tumors and lesions originate. Therefore, the tissue in the neoplastic field can be used to identify and study the earliest events in cancer progression [6,7]. This phenomenon has been examined in many cancers, including colon, lung, esophageal, ovarian, cervical, breast, prostate, and head and neck [7]. Colorectal cancer (CRC) presents a well-studied example of field cancerization due to its characteristic continuous epithelium which shares environmental influences. Many of the epigenetic [8,9], proteomic [10], and structural [11,12] alterations associated with CRC have been reported in the normal colonic mucosa adjacent to the tumor. Specifically, profound changes in the nuclear structure of cells in the microscopically normal rectal mucosa from patients with an adenoma or adenocarcinoma were suggested [13-15]. Moreover, these changes were correlated with the risk of recurrence of a colonic lesion [16]. These studies indicate that structural changes in the nuclear chromatin in the field of colon cancer represent a pre-neoplastic event. Therefore, understanding of the nuclear structure and how it reflects the process of malignant transformation is vital for the development of improved tools for cancer diagnosis and risk assessment.

One promising approach to quantify chromatin organization and function is through the spatial correlation of chromatin density distribution and, in particular, its fractal dimension [17-20]. The fractality of chromatin organization, validated by various methods [19-23], is the property of its self-similarity at different physical length scales. The fractal dimension of chromatin packing, in turn, is related to the amount of volume occupied by the surface of chromatin (a higher fractal dimension reflects a higher amount of exposed chromatin surface). Mathematically, a fractal medium is characterized by a power-law spatial correlation function, ~*r*^(*D*-3)^, with *D* being the fractal dimension of the medium. Reports show that the fractal dimension is increased in tumor cell nuclei. Moreover, the more aggressive the tumor, the less it resembles a mathematically ideal fractal [24,25]. Given the importance of chromatin structure for genome function, it is crucial to understand chromatin reorganization at the early stages of carcinogenesis. While nanoscale structural alterations in the field of CRC have been reported, these changes have not been visualized and identified until now due to the diffraction-limited resolution of optical techniques [13,15]. In order to further investigate premalignant chromatin structure, a technique with higher resolution is required.

In the present study, we take advantage of the nanoscale resolution of transmission electron microscopy (TEM) to investigate pre-microscopically detectable chromatin rearrangements in histologically normal-appearing cell nuclei in two models of early-stage CRC. We study pre-neoplastic chromatin rearrangements in human rectal cell nuclei from the field of CRC, as well as in animal colonic nuclei at a pre-malignant time point of the established azoxymethane (AOM)-injected rat model of CRC. We quantify the chromatin arrangement using approaches with two levels of complexity: 1) binary, where chromatin is separated into areas of dense heterochromatin and loose euchromatin, and 2) grey-scale, where the statistics of continuous chromatin density distribution is quantified via the spatial correlation function. We found significant and similar changes in the heterochromatin content, clumping and positioning in early and field carcinogenesis. Moreover, we show that these alterations correspond to the well-known hallmarks of cancer, but manifested at smaller, microscopically undetectable length scales. These results signify that the alterations in chromatin observed in the field of a tumor represent an early-stage event of carcinogenesis. We propose that the nanoscale nuclear abnormalities identified here can be employed as a biomarker for cancer prevention and diagnosis.

## Methods

### Subjects and samples

This study was conducted with the approval of the NorthShore University HealthSystem Institutional Review Board (IRB). Human biopsies were obtained from endoscopically normal rectal mucosa with an informed consent obtained from each subject prior to the procedure. Histopathologically all tissue samples appeared normal. Ten patient biopsies were used in this study, which included five normal and five from patients with adenomas (ranging in adenomatous polyp size from 2 to 10 mm). The biopsies were first placed in Karnovsky’s fixative for 2 weeks to preserve structure. The fixative consists of 0.1 M phosphate buffered solution containing 5% glutaraldehyde. Following standard protocol, the samples were stained with osmium tetraoxide (OsO4, commonly used to visualize DNA structure [26-28]), dehydrated, and then embedded in resin. Samples were then sectioned with an ultramicrotome to a thickness of 70 nm.

Animal procedures were performed at NorthShore University HealthSystem, with the approval of Institutional Animal Care and Use Committee (IACUC). Eighteen Fisher 344 rats (150–200 g; Harlan, Indianapolis, IN) were randomized to two weekly treatments of 15 mg/kg AOM (Midwest Research Institute, Kansas City, MO) or saline. Rats were euthanized at a premalignant time point, 10 weeks post injection, and necropsy was performed to confirm the absence of adenomas in the colon. To maintain good structural morphology, High Pressure Freezing (HPF) of animal colon samples was performed using a Leica EM-PACT2 high-pressure freezer at the Biological Imaging Facility (BIF) of Northwestern University. Automatic Freeze Substitution (AFS) was performed using a Leica AFS2 system. Samples were then embedded in Epon 812 resin (Electron Microscopy Sciences, Hatfield, PA) and thin-sectioned using Leica Ultracut S microtome into 90 nm sections onto copper grids.

### Image acquisition

**Figure 1:**
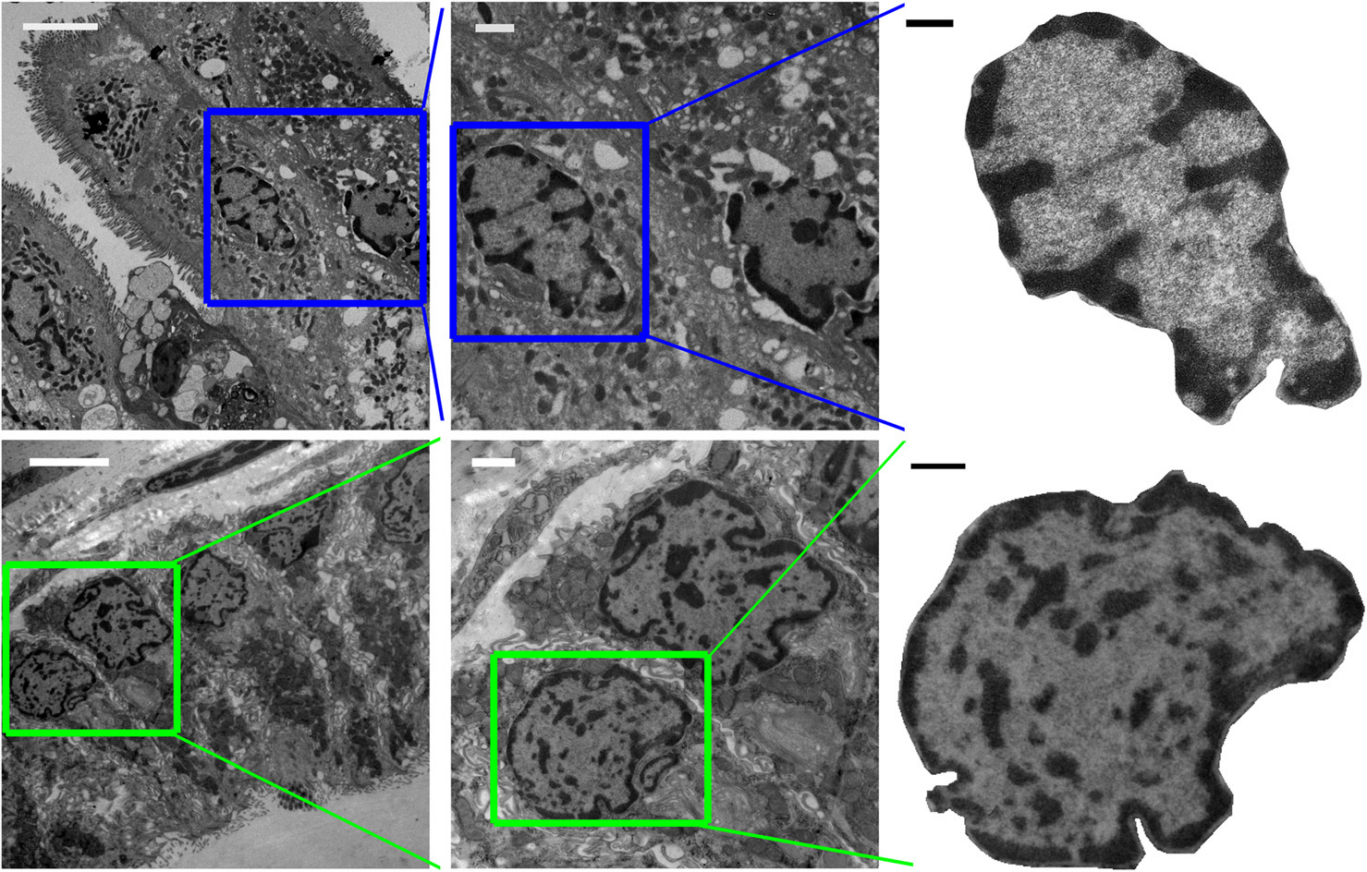
**Selection of nuclei from micrographs of human (top) and rat (bottom) colonic tissue.** Scale bars correspond to 4 microns (left), 1 micron (center), and 500 nm (right).

TEM micrographs for histologically normal rectal cells from control patients and those harboring a pre-cancerous adenoma elsewhere in the colon were obtained using a JEM-1400 (pixel size 7.8 nm). Images of animal colonic samples were collected using a JEOL 1230 and Advanced Microscopy Techniques imaging software at Northwestern University (pixel size 8.2 nm). While pixel resolution in the obtained micrographs was around 8 nm, due to the spherical aberrations and imperfect focusing the actual resolution of the obtained images from human samples was 39 nm (measured as the full-width half-maximum of the point-spread function of the imaging system). In the following animal study with improved image acquisition the resolution was 8.2 nm. Nuclei were manually selected from the tissue micrographs using Adobe Photoshop (example shown in Figure 1).

### Run length and heterochromatin percentage

Higher-order chromatin structure differentially regulates genomic loci through partitioning into active (euchromatin) and inactive (heterochromatin) domains (as reviewed in Ref. [29]). Changes in heterochromatin content and distribution have long been used as a marker for disease. Following standard TEM preparation and staining protocol, heterochromatic regions are heavily stained while euchromatic regions are lightly stained. Thus, nuclear TEM images were binarized to separate between these darkly-stained areas filled with the dense heterochromatin and the light euchromatin. For analysis, 1) the total amount of heterochromatin, and 2) the characteristic size (run length) of its clumps were measured. The first was calculated as the percentage of the nuclear area occupied by heterochromatin. The second was quantified via a parameter termed run length, which is defined as the average number of connected, consecutive pixels with values corresponding to heterochromatin. Each micrograph was scanned in horizontal and vertical directions to record the run lengths of all heterochromatin occurrences. The average of all recorded values was defined as the heterochromatin run length of the image.

### Distribution with respect to periphery

**Figure 2:**
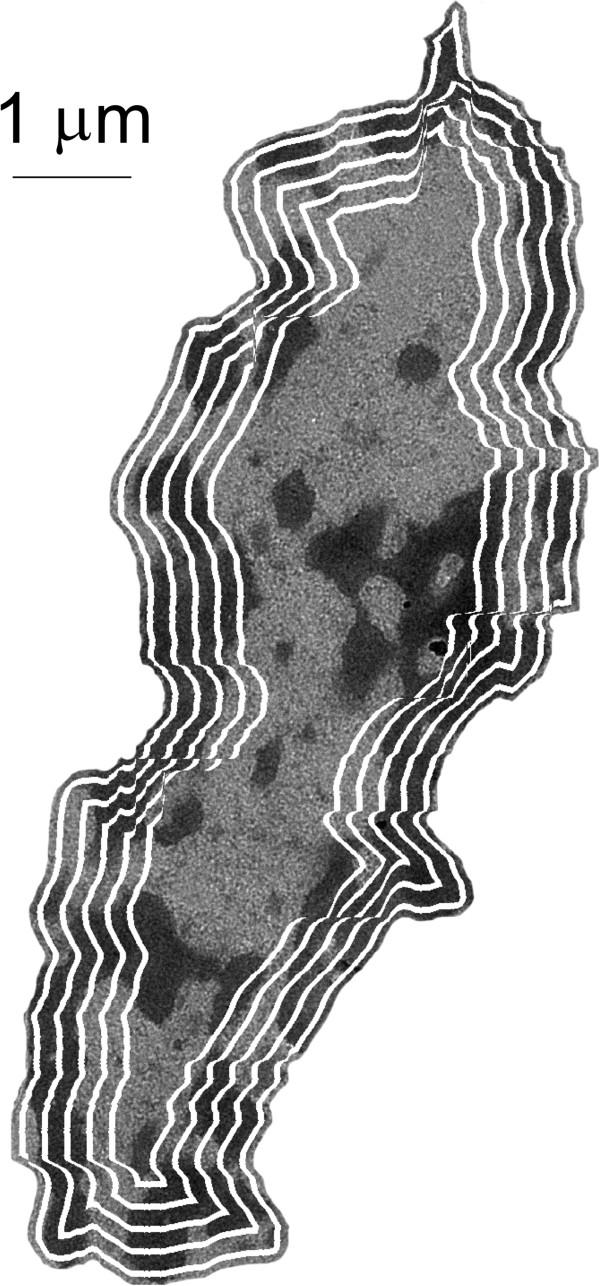
**Selection of nuclear periphery.** TEM image of a nucleus, where white lines illustrate areas equidistant from the nuclear envelope.

Most normal cells exhibit a distinct region of heterochromatin located around the nuclear periphery, while cancerous cells exhibit a loss of this heterochromatin border. To quantify the chromatin distribution with regards to the nuclear periphery, we measured the percentage of the total heterochromatin located within a specific distance from the nuclear envelope. Nuclear space was separated into ribbon-like areas of equal distance from the nuclear periphery (Figure 2). The width of each ribbon in nuclei from human samples was 37.5 nm, and 16.4 nm from rat samples. The fraction of heterochromatin located within each ribbon (relative to the total heterochromatin content) was measured. Then, the percentage of heterochromatin as a function of the distance from its location to the nuclear envelope was calculated. Nucleoli were not considered for analysis given their distinct function in ribosomal RNA transcription and assembly. Meanwhile, the heterochromatin region at the surface of nucleoli is another key characteristic of normal cell nuclei that is lost in cancerous cells. Thus, we classified heterochromatin located around the nucleolar surface together with that located around the nuclear periphery.

### Spatial correlation function

**Figure 3:**
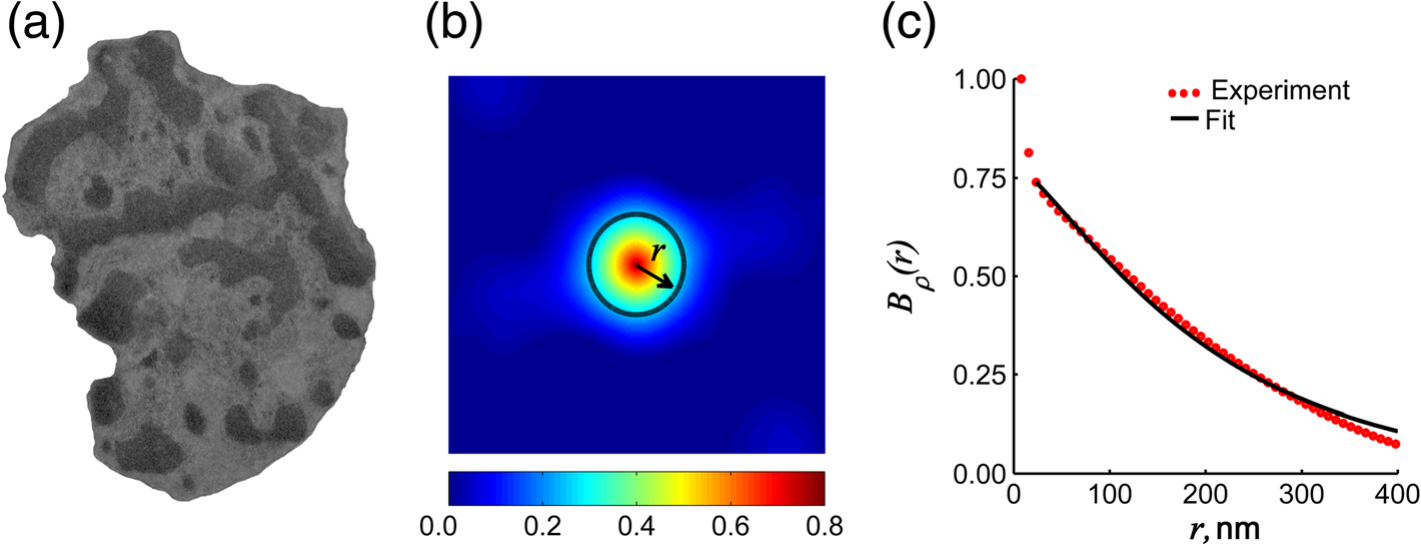
**Calculation of spatial correlation function. (a)** Gray-scale TEM image of an example nucleus from a human sample, **(b)** color-coded map of 2-D spatial correlation function obtained from it. Black dashed circle outlines data points corresponding to the same separation r, and **(c)** 1-D spatial correlation function calculated by averaging data for each separation r (red circles) and the analytical correlation function fitted to the experimental (black solid line).

Gray-scale TEM images of nuclei were analyzed using MATLAB (Mathworks) computing software. To obtain an image of chromatin density fluctuations, the mean grey-scale value was subtracted from the image. Then, the two- dimensional correlation function of the spatial distribution of chromatin was obtained using the Wiener-Khinchine relation as:

(1) $$B_{\rho }\left(x,y\right)=F^{-1}\left\{\left|F\right)\left(\rho_{\Delta }\left(x,y\right)\right)\left|{}^{2}\right)\right\},$$

where *F*^-1^ and *F* are the inverse and the direct two-dimensional fast Fourier transforms, performed via the built-in Matlab function fftn, and *ρ*_*Δ*_ is the fluctuating part of the density of the nuclear material. Then, a rotational average of *B*_ρ_ (*x,y*) was taken to obtain the one-dimensional *B*_ρ_ (*r*) representing the degree of mass density correlation as a function of separation *r* (Figure 3b). To account for the sample-to-sample variability in image contrast due to differences in the depth of staining, *B*_ρ_ (*r*) was normalized to 1 at *r = r*_*min*_, where *r*_*min*_ is the resolution of the image. Additionally, to remove the effect of nuclear shape and size, *B*_ρ_ (*r*) was truncated at *r = r*_*max*_, where *r*_*max*_ represents the length scale at which the spatial correlation decreases to a negligible level, i.e. *B*_ρ_ (*r*_*max*_) = 0.02. The correlation functions between *r*_*min*_ and *r*_*max*_, obtained from every image were fitted to a three-parameter Whittle-Matern family of correlation functions of the following functional form:

(2) $$B_{\rho }\left(r\right)=A_{\rho }{\left(\frac{r}{l_{c}}\right)}^{\frac{D-3}{2}}K_{\frac{D-3}{2}}\left(\frac{r}{l_{c}}\right),$$

where $K_{\frac{D-3}{2}}(\cdot )$ is the modified Bessel function of the second kind of the order $\left(\frac{D-3}{2}\right)$, *l*_c_ is the characteristic length of heterogeneity of the nuclear material, *A*_*ρ*_ is the chromatin density fluctuation amplitude, and *D* determines the shape of the distribution.

While *A*_*ρ*_ and *l*_*c*_ account for the variability in depth of staining and image size, the third parameter of the correlation function, *D*, uniquely quantifies the *shape* of the mass density spatial correlation function. By fitting the appropriate value of *D*, we are able to quantify 1) the relative length scale composition of the nucleus (increase in *D* implies a shallower decay of correlation function and, therefore, higher relative presence of larger length scales), 2) the functional forms of spatial correlation of mass distribution (e.g. Gaussian at *D → ∞*, decaying exponential at *D* = 4, stretched exponential at 3 < *D* < 4 and power law at *D* < 3). Thus, *D* delivers an excellent qualitative and quantitative measure of the experimentally obtained spatial correlation of chromatin density.

To fit the experimentally obtained *B*_*ρ*_(r), an array of Whittle-Matern family correlation functions was created with values of *D* ranging from 2 to 6 and *l*_*c*_ ranging from 0.66*r*_*max*_ to *r*_*max*_. The values of *D* and *l*_*c*_, which yield the best agreement (evaluated via *r*-squared values of the fit) between the experimental and fitted curves, were calculated for every image.

## Results

**Figure 4:**
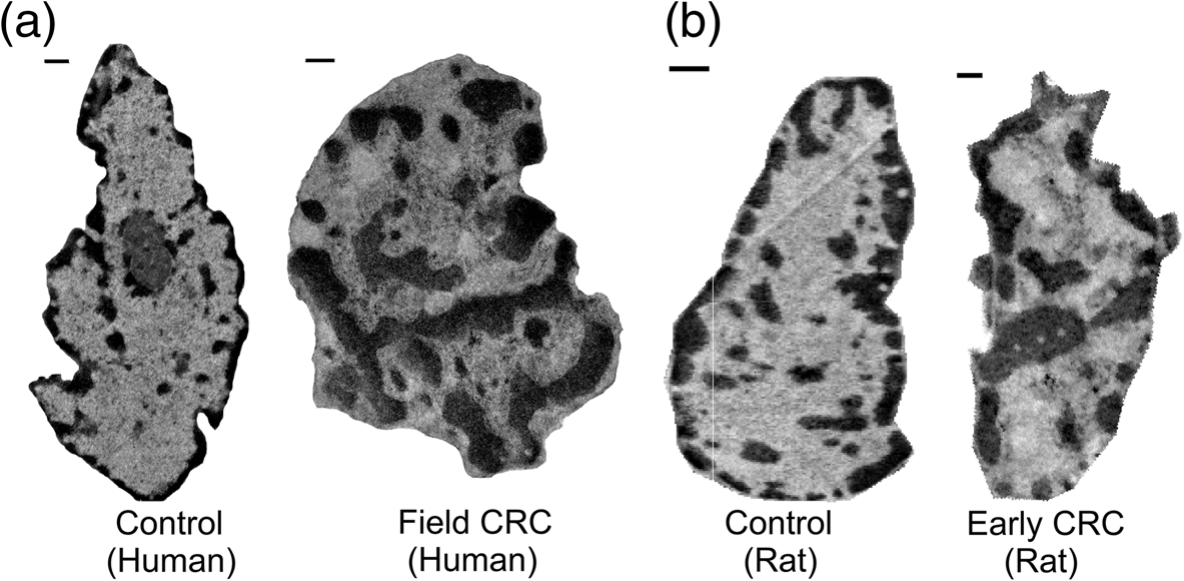
**Example TEM micrographs. (a)** Histologically normal rectal cell nuclei from control patients and those harboring a pre-cancerous adenoma elsewhere in the colon, representing field CRC. Scale bars correspond to 500 nm. **(b)** Histologically normal colonic cell nuclei from control rats and those treated with azoxymethane for 10 weeks (premalignant time point), representing early CRC. Scale bars correspond to 250 nm.

We obtained 36 control and 29 field CRC TEM micrographs from patient samples as well as 107 control and 51 early CRC micrographs from animal samples (representative images shown in Figure 4). While various definitions of chromatin compartments have been proposed [30-33], we here followed the classical cytological partitioning of chromatin that is based on purely morphological criteria.

### Binary quantification of chromatin compaction

**Figure 5:**
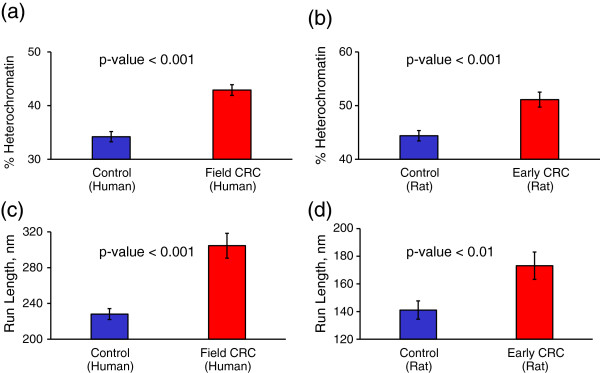
**Heterochromatin percentage and run length. (a)** Average % heterochromatin in rectal nuclei of healthy human patients (control) and those horboring tumor elsewhere in the colon (field CRC). **(b)** Average % heterochromatin in colonic nuclei of rats treated with saline (control) and azoxymethane at a pre-malignant time point (early CRC). Average run length of heterochromatin in **(c)** human and **(d)** rat samples. Error bars correspond to the standard error between images.

We first described chromatin organization in terms of its two conformations: highly condensed heterochromatin and relatively loose euchromatin. Heterochromatin is largely considered to be transcriptionally silent and is localized primarily to the nuclear periphery, while euchromatin is the active form of chromatin and is extended throughout the nucleus. In both models of early CRC we observed a significantly increased characteristic size of heterochromatin aggregates, quantified via run length (Figure 5c,d), which is consistent with the karyometric study from Ref. [15]. In patient samples from the field of CRC the run length increased from 228 nm to 305 nm (Figure 5c), and in rat samples of early CRC the run length increased from 141 to 173 nm (Figure 5d). The average sizes of nuclei of patient samples were 60% larger than that of rat samples, which explains the difference in the run length values between two models. At the same time, there was no difference in nuclear area between controls and cases within either model (p > 0.5 in both patient and rat models). Additionally, we established that not only the characteristic size, but the total percentage of heterochromatin is significantly increased in both models of early-stage CRC (from 34.2% to 42.9% in humans and from 44.4% to 51.1% in rats, Figure 5a,b).

**Figure 6:**
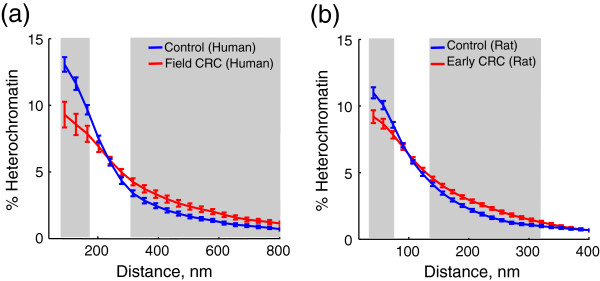
**Heterochromatin distribution with regards to nuclear periphery.** Results shown for the models of **(a)** human control and field CRC samples and **(b)** rat control and early CRC samples**.** Grey background indicates length scales at which the difference between the two groups is statistically significant (p < 0.05). Error bars correspond to the standard error between images.

Next, we investigated the location of condensed chromatin areas in the nucleus. The 3D chromatin structure of most normal cells is such that the chromatin fibers positioned towards the nuclear interior are characterized as: 1) gene-rich (from a 1D genome perspective), 2) actively transcribed (from a nuclear function perspective), and 3) more open/decondensed (from a physics perspective) [34-39]. Accordingly, a distinct region of gene-poor, transcriptionally inactive and highly condensed heterochromatin tends to be located towards the nuclear periphery [40,41]. Upon analysis of the TEM micrographs, we determined that the heterochromatin distribution relative to the nuclear periphery was substantially altered in both studied models of early CRC. We observed a statistically significant decrease in the amount of heterochromatin located at the nuclear periphery and a statistically significant increase in the amount located in the nuclear interior (Figure 6). These findings have important implications: heterochromatin plays major roles in gene silencing, chromosome segregation and genomic integrity, and its expansion across chromatin domains often leads to epigenetic silencing of nearby genes [30,31].

### Grey-scale quantification of chromatin compaction

**Figure 7:**
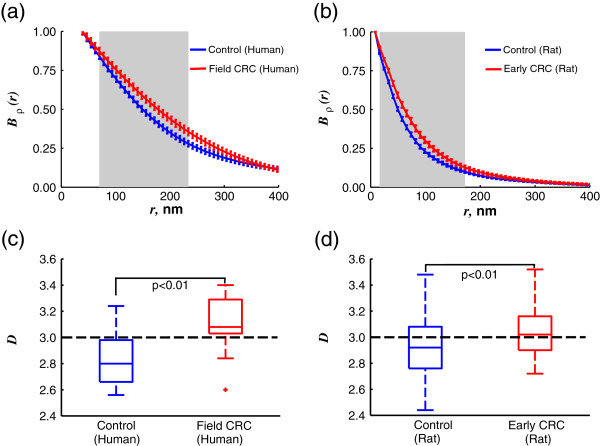
**Quantification of chromatin mass density spatial correlation function *B***_**ρ**_**(*r*).** Average *B*_ρ_(*r*) of **(a)** human control and field CRC samples and **(b)** rat control and early CRC samples. *B*_ρ_(*r*) is not defined at length-scales below the image resolution (39 nm for human and 8.2 nm for rat samples). Grey background indicates length scales at which the difference between the two groups is statistically significant (p < 0.05), error bars represent standard error between images. Boxplots shown for all values of *D* corresponding to correlation functions of **(c)** human and **(d)** rat samples.

Finally, we exploited the grey-scale information of the TEM micrographs to characterize the spatial heterogeneity of chromatin distribution in further detail. We quantified the relative magnitudes and length scales of all spatial fluctuations in the degree of chromatin compaction via its spatial correlation function *B*_ρ_(r). Upon comparison of chromatin density correlation between the controls and cases representing early-stage cancer, we have found a significant difference in chromatin distribution at subdiffractional length scales (Figure 7a,b).

We also quantified the shape of the chromatin correlation function by fitting the experimentally measured *B*_ρ_(r) from every micrograph to the family of Whittle-Matern correlation functions. This analysis revealed that 1) there is a significant increase in the width of correlation function, and, therefore, in the dominance of larger length scales in pre-cancerous nuclei (quantified via *D*); and 2) the type of spatial correlation function changes from fractal in the case of healthy nuclei (*D* < 3, in agreement with [19-23]) to a stretched exponential in precancerous cell nuclei (Figure 7). While the first finding supports a previously reported increase in run length, the second finding gives a different perspective on describing both nuclear structure and dynamics. The change in the type of spatial correlation has major implications in the physical forces and size-selectivity that regulate nuclear function. Interestingly, this transition in the type of spatial correlation function from fractal to a stretched exponential observed in cells was previously described in malignant ovarian tissue reorganization [42].

## Discussion

Proper higher-order three-dimensional organization of chromatin is crucial for normal cell function, influencing gene expression and DNA replication and repair. Abnormalities in chromatin organization have been described in a variety of diseases [1,2]. While abnormal chromatin aggregation and clumping observed by an optical microscope is a well-studied hallmark of carcinogenesis, little is known about changes in nuclear organization that precede this stage of cancer progression. This is largely due to the diffraction limit of light microscopy used in conventional histology, which is unable to characterize intracellular structures smaller than 250 nm. Thus, to identify the pre-microscopically detectable abnormalities in higher-order chromatin structure at the earliest stages of carcinogenesis, imaging technology with a greater resolution is needed. Here, we use nanoscale-resolution TEM to study the chromatin organization in microscopically normal-appearing cell nuclei that may be undergoing early-stage tumorigenic alterations. We provide quantitative and qualitative descriptions of the pre-neoplastic nuclear ultrastructural (i.e. microscopically indiscernible) changes in the field of CRC in humans, as well as in early premalignant-stage CRC in AOM-treated rats.

The analysis of heterochromatin distribution revealed an increase in heterochromatin content and clump size, as well as a loss of its characteristic peripheral positioning in both models of early cancer (Figures 4, and 5). Note that these heterochromatin rearrangements, also identified as chromatin coarsening, are a well-known feature of cancer when resolved by an optical microscope (for review, see [3]). We have also observed profound changes in the spatial correlation of chromatin density at length scales smaller than 250 nm (Figure 7a,b), which remarkably coincides with the diffraction limit of optical microscopes. Thus, we established that in the series of sequential events involved in malignant transformation, *nanoscale* chromatin compaction and rearrangement precedes *microscale* chromatin compaction and rearrangement.

Additionally, we found a change in the type of chromatin density correlation from fractal in normal nuclei to a stretched exponential in the pre-cancerous nuclei (Figure 7). This important finding has implications beyond the purely structural. The fractal nature of chromatin distribution in normal cells, or self-similarity at different length scales, ensures that the diffusion rates for transcription factors are independent of their size. Meanwhile, the stretched exponential distribution is described by a characteristic length scale, implying preferred diffusion of particular-size proteins. Thus, the change in the type of chromatin spatial correlation may alter the diffusion of macromolecules inside the nucleoplasm and hence the chromatin’s accessibility to transcription factors. We conclude that the extensive structural chromatin alterations reported here represent the earliest known events in carcinogenesis which likely drive the changes in gene expression during neoplastic transformation.

The novel characterization of premalignant nuclear architecture reported here establishes new biomarkers of early tumorigenesis. This opens the door for a set of new technologies to develop and perform tests for the diagnosis and risk stratification of cancer. From a clinical perspective, TEM-based methods are impractical, as they are time consuming, costly, and rely on heavy processing of the sample. Meanwhile, optical techniques present distinct advantages of time- and cost-effectiveness, and can be performed by primary care physicians, complementing the pathological examination and enhancing diagnosis through implementation of an automated analysis. The development of ultrastructural biomarkers can aid in cancer diagnosis when detected by scattering-based techniques, such as optical coherence microscopy, light-scattering spectroscopy, confocal light absorption and scattering spectroscopy, and partial wave spectroscopic microscopy [43-45].

Several potentially important measures of nuclear structure could not be quantified in the present study due to its technological limitations, as follows. The precise shape of the nucleus, known to be altered in cancer, could not be characterized due to the dehydration involved in sample fixation protocol that is likely to distort the native shape of nuclei. Nuclear size and total number of nucleoli were not measured as they are highly dependent on the sample sectioning. The absolute values of chromatin mass density variations could not be determined due to the employed staining. Finally, the imaging resolution of the employed TEM instrumentation did not allow us to resolve structural changes at length scales smaller than the chromatin fiber. Future studies involving advanced electron microscopy techniques with improved spatial resolution and using protocols that better preserve native chromatin structure will provide new insights into pre-neoplastic transformation of the nuclear structure.

Furthermore, to fully understand the changes in genomic activity resulting from the established structural alterations, it is important to note that the transcriptional status of an individual gene is not always determined by its nuclear location and the degree of local chromatin condensation. Despite the correlation between gene density, transcriptional activity, and nuclear positioning, there are also many reports demonstrating deviations from this rule. For example, heterochromatin is not always transcriptionally silent [46-48]; active genes can be present in the periphery of the nucleus; and inactive genes can be located in the interior [49-51]. Therefore, the effect of early tumorigenic structural changes in 3D chromatin structure on genome function needs to be further investigated. This may be achieved by integrating the subdiffractional sensitivity of the interferometric spectroscopy of scattered light [52] and the targeted enhancement of optical contrast [53] by careful selection of transcription activity-related protein markers [32].

## Conclusions

In this manuscript we identify significant quantitative and qualitative changes in chromatin distribution in field and early carcinogenesis. We confirm that the ultrastructural field effect changes of nuclear organization represent the initial steps that lead to the development of well-known, microscopically detectable hallmarks of cancer. We conclude that the established alterations in higher-order chromatin structure is a crucial early event in tumorigenesis. Identifying pre-neoplastic changes in the tumorigenic field is a promising area of research in order to develop novel tools for cancer prediction and diagnosis.

## Abbreviations

CRC: Colorectal cancer; TEM: Transmission electron microcopy; AOM: Azoxymethane.

## Competing interests

The authors declare that they have no competing interests.

## Authors’ contributions

LC developed and performed the grey-scale image analysis and wrote the manuscript. YSC carried out the TEM imaging, participated in the image analysis and helped write the manuscript. HS and CW designed and carried out the binary image analysis. HKR and VB co-conceived the project and managed its design and coordination. MDC and RKW designed the animal study and acquired tissue specimens. MJG and LKB designed the human study and acquired tissue specimens. All authors read and approved the final manuscript.

## Pre-publication history

The pre-publication history for this paper can be accessed here:

http://www.biomedcentral.com/1471-2407/14/189/prepub
